# Supplementary material for: Category-biased patches encircle core domain-general regions in the human lateral prefrontal cortex
Source: bioRxiv. 2025 May 3:2025.01.16.633461. Originally published 2025 Jan 16. Preprint. [Version 2] doi: 10.1101/2025.01.16.633461 (PMC11761636; doi:10.1101/2025.01.16.633461)
Supplement: 1 [file NIHPP2025.01.16.633461V2-supplement-1.pdf]

bioRxiv preprint doi: https://doi.org/10.1101/2025.01.16.633461; this version posted May 3, 2025. The copyright holder for this preprint (which was not certified by peer review) is the author/funder, who has granted bioRxiv a license to display the preprint in perpetuity. It is made available under aCC-BY 4.0 International license.

Supplementary Figures

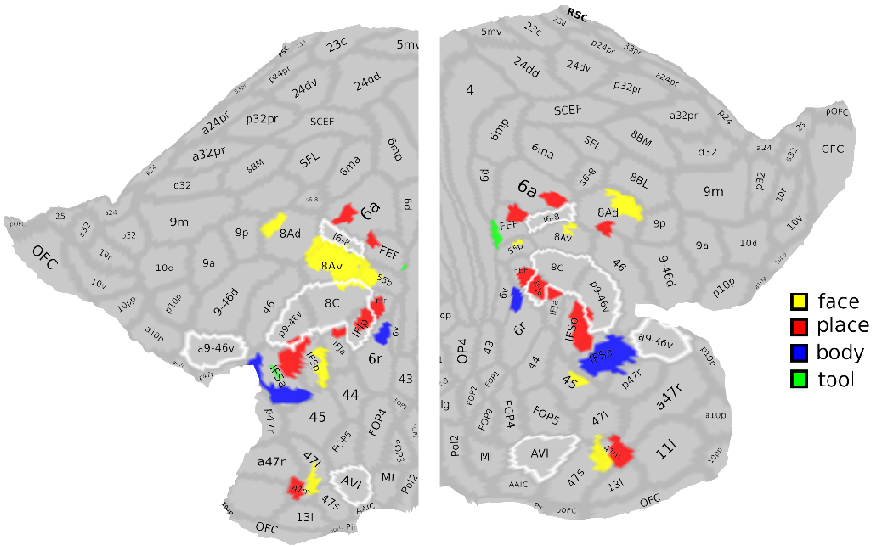

Supplementary Figure 1. Category-biased patches in the HCP dataset from **Figure 1** overlaid on the areal labels of the HCP MMP1.0 (Glasser et al., 2016). Data in this figure are available at https://balsa.wustl.edu/3q0lx

bioRxiv preprint doi: https://doi.org/10.1101/2025.01.16.633461; this version posted May 3, 2025. The copyright holder for this preprint (which was not certified by peer review) is the author/funder, who has granted bioRxiv a license to display the preprint in perpetuity. It is made available under aCC-BY 4.0 International license.

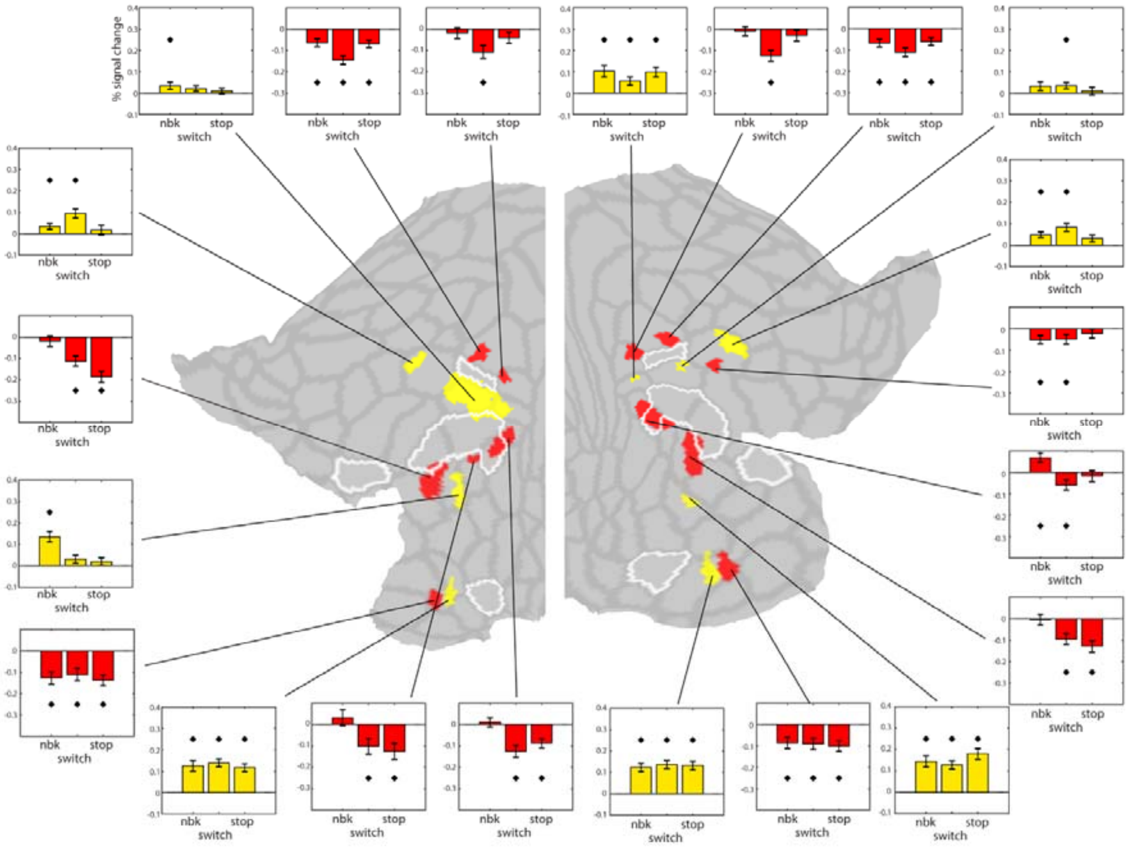

Supplementary Figure 2. Estimated response (face minus place) for each EF task [n-back (nbk), switch, stop] to the HCP's n-back defined face and place ROIs. Core MD regions (white borders) are based on the definition in (Assem et al., 2020). * indicates p<0.05 Bonferroni corrected within each patch for 3 tasks.
